# Supplementary material for: MetaRibo-Seq measures translation in microbiomes
Source: Nat Commun. 2020 Jun 29;11:3268. doi: 10.1038/s41467-020-17081-z (PMC7324362; doi:10.1038/s41467-020-17081-z)
Supplement: Supplementary file 10 — Supplementary Data 7 [file 41467_2020_17081_MOESM10_ESM.zip › File2/Confidence_High_Taxonomy/85573_out.krona.html]

Javascript must be enabled to view this page.

members
magnitude
magnitudeUnassigned
count
unassigned
taxon
rank

85573\_out

9

8

SRS014287\_contig\_number\_contig-100\_22171.62028SRS024625\_contig\_number\_contig-100\_23816.23816SRS053335\_contig\_number\_contig-100\_23924.62487SRS053398\_contig\_number\_contig-100\_34378.73253SRS062654\_contig\_number\_contig-100\_35863.78628SRS077552\_contig\_number\_contig-100\_38372.38372SRS1055034\_contig\_number\_contig-100\_26904.26905SRS893270\_contig\_number\_contig-100\_38068.78787

2
1
superkingdom

phylum
976
1

class
1
117743

order
200644
1

1
49546
family

290174
1
genus

SRS143085\_contig\_number\_contig-100\_6881.6882
